# Pachychoroid: current concepts on clinical features and pathogenesis

**DOI:** 10.1007/s00417-020-04940-0

**Published:** 2020-10-15

**Authors:** Veronica Castro-Navarro, Francine Behar-Cohen, Woohyok Chang, Antonia M. Joussen, Timothy Y. Y. Lai, Rafael Navarro, Ian Pearce, Yasuo Yanagi, Annabelle A. Okada

**Affiliations:** 1 Hospital General Universitario de Valencia, Valencia, Spain; 2 Hôpital Cochin, Assistance Publique Hôpitaux de Paris, Ophtalmopole, Paris, France; 3 Centre de Recherche des Cordeliers UMRS1138, INSERM, Paris, France; 4 Chang’s Retina Center, Daegu, South Korea; 5 Charité–University Medicine Berlin, Berlin, Germany; 6 Department of Ophthalmology & Visual Sciences, The Chinese University of Hong Kong, Hong Kong, China; 7 Institute of Ocular Microsurgery, Barcelona, Spain; 8 Royal Liverpool University Hospital, Liverpool, UK; 9 Department of Ophthalmology, Asahikawa Medical University, Asahikawa, Japan; 10 Singapore National Eye Centre, Singapore, Singapore; 11 Department of Ophthalmology, Kyorin University School of Medicine, Tokyo, Japan

**Keywords:** Pachychoroid, Pachychoroid pigment epitheliopathy, Pachychoroid neovasculopathy, Focal choroidal excavation, Peripapillary pachychoroid syndrome, Pachydrusen

## Abstract

**Purpose:**

The term “pachychoroid” refers to a newly described phenotype in which functional and structural choroidal changes are thought to play a key pathogenic role in a spectrum of related retinal disorders. A more detailed understanding of how the choroid is involved within this spectrum and a better knowledge of the most relevant clinical signs of the pachychoroid phenotype are important to differentiate these disorders from other retinal conditions. Our objectives are to provide a literature review of pachychoroid and the commonalities that may be present across pathologies included in the spectrum, and to provide details on the examination, monitoring, and management of these disorders.

**Methods:**

We searched the PubMed web platform to identify relevant studies using the following keywords: pachychoroid, pachychoroid pigment epitheliopathy, pachychoroid neovasculopathy, aneurysmal type 1 neovascularization, focal choroidal excavation, peripapillary pachychoroid syndrome, vasculopathy pachysclera, pachychoroid geographic atrophy, and pachydrusen. We selected 157 publications and identified the most important features related to pachychoroid.

**Results:**

The presence of hypertrophic or congested vessels in the choroid, not thickened choroid per se, under an area of reduced or absent choriocapillaris in the posterior pole seems to be the most salient feature of pachychoroid. However, other qualitative/quantitative features are needed to differentiate the uncomplicated pachychoroid from the pathological pachychoroid clinical spectrum, which may be associated with exudation, neovascularization, and/or retinal pigment epithelium and photoreceptor atrophy.

**Conclusions:**

The most salient feature of pachychoroid appears to be the presence of large vessels under an area of reduced or absent choriocapillaris. Knowledge of the features and pathogenesis of the different disorders in the pachychoroid spectrum may assist in the management of patients.

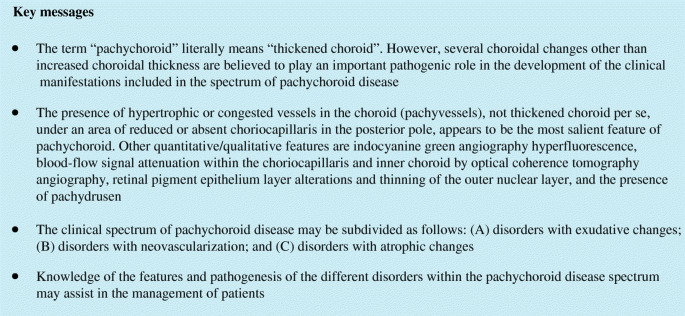

## Introduction

The term “pachychoroid” refers to a newly described phenotype in which functional and structural choroidal changes are thought to play a key pathogenic role in a spectrum of related retinal disorders, the “pachychoroid spectrum” [1]. Advances in imaging technology over the past decade have led to new insights and a better understanding of changes within the choroid in disorders previously identified predominantly by their retinal manifestations [2]. Several disorders, such as central serous chorioretinopathy (CSC), polypoidal choroidal vasculopathy (PCV), pachychoroid neovasculopathy (PNV), and pachychoroid pigment epitheliopathy (PPE), overlap within the pachychoroid spectrum. However, the lack of a consensus on various phenotypes can lead to these disorders being categorized together with neovascular age-related macular degeneration (nAMD), and in some cases, PCV is included in clinical trials for nAMD, despite having different clinical characteristics, natural history, and response to treatment [3]. In order to avoid such miscategorization, an understanding of how the choroid is involved within this spectrum and a better knowledge of the most relevant clinical signs of the pachychoroid phenotype are important to differentiate these disorders from other chorioretinal conditions.

The objectives of this review article are to provide a consensus on the definition of pachychoroid and the commonalities that may be present within the pathologies included in this spectrum, as well as to provide details on the examination, monitoring, and management of these disorders.

## Methods

This article was based on a review of the literature and a consensus among retinal experts who are members of the Vision Academy, an international group of retinal physicians who work together to share existing skills and knowledge and provide collective recommendations on clinical challenges in areas where there is a lack of conclusive evidence in the literature (www.visionacademy.org). The Vision Academy is fully sponsored by Bayer.

A literature search was performed using the PubMed web platform to identify relevant publications using the following keywords: pachychoroid, pachychoroid pigment epitheliopathy, pachychoroid neovasculopathy, aneurysmal type 1 neovascularization, focal choroidal excavation, peripapillary pachychoroid syndrome, vasculopathy pachysclera, pachychoroid geographic atrophy, and pachydrusen. More than 150 publications were reviewed to identify key studies showing important features related to pachychoroid.

## Epidemiology

Information on the prevalence of pachychoroid spectrum disorders is scarce and the lack of a consensus on their definition makes it harder to conduct epidemiological studies that encompass the whole clinical spectrum. However, some important epidemiological differences have been described between exudative AMD versus PCV or CSC.

Numerous population-based studies and other data on the epidemiology of AMD have been reported around the world, with the results of some studies suggesting racial or ethnic differences in disease prevalence. An analysis of 129,664 individuals (aged 30–97 years), with 12,727 cases from 39 studies, showed global prevalence rates of early-, late-, and any-stage AMD of 8.01% (95% confidence interval [CI] 3.98, 15.49), 0.37% (95% CI 0.18, 0.77), and 8.69% (95% CI 4.26, 17.40), respectively [4]. A higher prevalence of early- and any-stage AMD in Europeans than in Asians was found (11.2% vs. 6.8% and 12.3% vs. 7.4%, respectively), and early-, late-, and any-stage AMD is more prevalent in Europeans than Africans, with no difference in prevalence between Asians and Africans [4].

In contrast to AMD, there are few robust data on the epidemiology of PCV, and accurate estimates of PCV prevalence from population-based studies have been difficult to obtain due to the inherent difficulty of diagnosing the disorder with certainty from fundus photos alone. As such, PCV prevalence has been estimated based on hospital- or clinic-based cross-sectional studies in 22.3–61.6% of Asians and 8–13% of Caucasians who present with presumed exudative AMD [5]. In the Hisayama Study, the characteristics of PCV were determined by fundus examination or based on findings from indocyanine green angiography (ICGA) and fluorescein angiography (FA). This study, which was performed in 2663 residents of Hisayama, Japan, aged ≥ 50 years, demonstrated that the prevalence of PCV was around 30.3% among the study participants with late AMD, which was higher than in Caucasians in Western countries [6]. The study also found that male gender and smoking habits were significant risk factors for developing PCV. The Beijing Eye Study was the first population-based study that attempted to estimate the prevalence of PCV using a combination of clinical and optical coherence tomography (OCT) diagnostic criteria [7]. Among 3468 subjects aged ≥ 50 years, PCV was diagnosed in 18 eyes (prevalence rate, 0.30–1.0%; 95% CI 0.1%, 0.4%), out of 17 subjects with a mean age of 74.5 ± 7.5 years (range, 59–87 years) and no gender predominance. The eyes with PCV were compared with the contralateral unaffected eyes, showing no differences in refractive error, axial length, or intraocular pressure; however, subfoveal choroidal thickness was significantly thicker in the affected eyes than in the contralateral eyes (275 ± 90 μm vs. 210 ± 92 μm; *p* = 0.02) or eyes with exudative AMD. This may imply that the morphologic difference between the disorders may be a surrogate for differences in the pathophysiology, and may explain differences in treatment outcomes observed with PCV as opposed to exudative AMD [7].

The Beijing Eye Study also provides information on CSC [8], a common retinal disease that has been associated with numerous risk factors. To date, most of the prevalence data available on CSC have been obtained from hospital-based studies, which are limited in terms of patient selection, and only a few studies have reported the prevalence and incidence of CSC and associated factors. Kitzmann et al. [9] performed a population-based, retrospective, cohort and case-control study to examine the incidence of CSC in Olmsted County, MN, USA, from 1980 to 2002. They found a mean annual age-adjusted incidence rate per 100,000 of 9.9 (95% CI 7.4, 12.4) for men and 1.7 (95% CI 0.7, 2.7) for women. Similarly, the Beijing Eye Study revealed a prevalence of CSC of 0.31% per subject in patients with a mean age of 55.2 ± 4.0 years, with a significantly higher proportion of men affected. CSC-affected eyes also had thicker choroids than in an age- and gender-adjusted control population-based group [8].

## Pachychoroid: quantitative and qualitative features

**Fig. 1:**
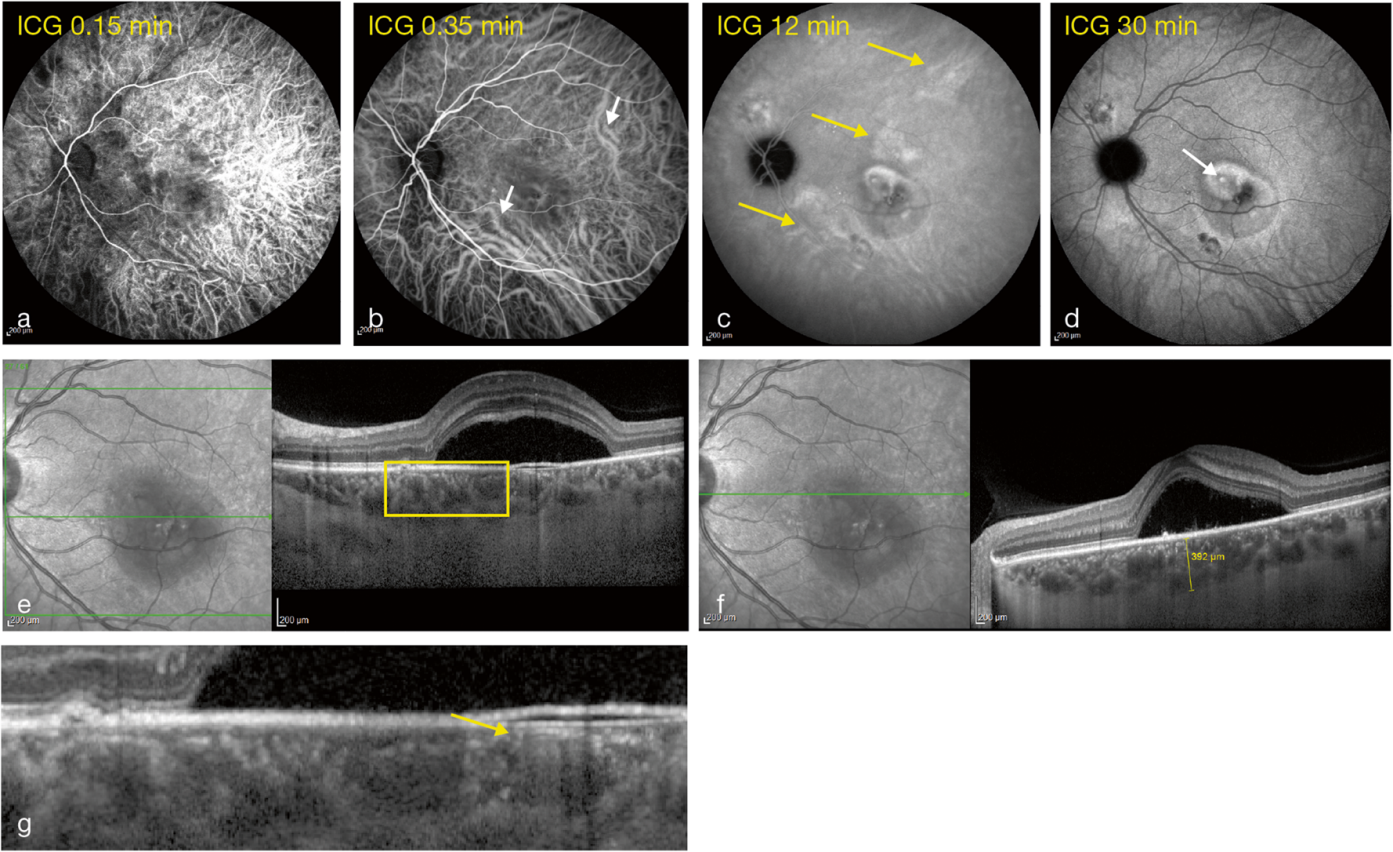
Images showing the left eye of a patient with CSC with mid-phase hyperfluorescence on ICGA (**a**–**d**). Pachyvessels (**e**). Increased choroidal thickening on OCT (**f**) with choriocapillaris thickness decrease (**g**) CSC, central serous chorioretinopathy; ICGA, indocyanine green angiography; OCT, optical coherence tomography

Comparisons of anatomical differences between PCV, CSC, and AMD in epidemiological studies have suggested differences in the pathophysiology of the disorders, where increased choroidal thickness appears to be a prevalent factor [7, 8]. However, several choroidal changes other than increased choroidal thickness are believed to play an important pathogenic role in the development of the clinical manifestations that reside within pachychoroid disease (Table 1). The term “uncomplicated pachychoroid” has been used to describe eyes with choroidal hyperpermeability, focal or diffuse congestion, or an increase in choroidal thickness and dilated choroidal vessels (pachyvessels) with reduced overlying choriocapillaris [10]. The presence of these features plus other quantitative or qualitative features of the pachychoroid spectrum may suggest that the pachychoroid pathogenic process has emerged and that a disorder falls within the “complicated or pathological” pachychoroid clinical spectrum, where overlapping features [19] and progression from one disorder to another are frequently observed. Key features seen in the eyes of patients with CSC are shown in Fig. 1.

**Table 1:** List of key clinical findings of the pachychroid spectrum of eye diseases

	Uncomplicated pachychoroid [10, 11]	Complicated or pathological pachychoroid					
		Those with exudative changes			Those with neovascularization		Those with atrophy
		FCE [12, 13]	CSC [1, 14–16]	PPS [11]	PNV [3]	PCV [17]	PPE [1, 14, 18]
Funduscopic appearance	N/A	• Normal fundus appearance or nonspecific pigmentary changes or indistinct yellow-whitish spots in an area of reduced fundus tessellation		• Normal fundus appearance or choroidal folds or disc edema	• Reduced fundus tessellation	• Reddish-orange nodular structures beneath the retina • Serous neurosensory detachment • Submacular hemorrhage • Serous or hemorrhagic PED, or both	• Reduced fundus tessellation • Minimal or absent choroidal vascular markings on clinical and drusenoid RPE changes and/or small PEDs
OCT	• Focal or diffuse increase of choroidal thickness • Dilated Haller’s layer vessels (termed “pachyvessels”) with thinning of the overlying inner choroid	• One or more focal excavations in the choroidal layer • Two patterns: a) nonconforming FCE and b) conforming FCE	• Serous retinal detachment • Serous PED • Focal defects of the RPE • Elongated photoreceptor outer segments • Outer retinal atrophy (disruption to EZ, thinning of ONL) • Cystoid macular degeneration in chronic CSC	• Thickened choroid preferentially in the nasal macular region • Intraretinal fluid and cysts extending from the nasal and temporal disc margin • Atrophy of the RPE, EZ, and ELM resulting in choroidal signal hypertransmission at the optic disc margin • Varying amounts of subretinal fluid	• Presence of type 1 CNV • Double layer sign • RPE abnormality independent of CNV lesion • Dilated Haller’s layer vessel with obliteration of choriocapillaris underneath the CNV	• Type 1 CNV with aneurysmal lesions • Double layer sign • PED	• Absence of macular edema and subretinal fluid • Small PEDs • Outer retinal atrophy (thinning of ONL)
OCT-A	N/A	• No evidence of CNV	• No evidence of CNV • Inner choroidal flow signal attenuation zones, mostly related with pachyvessels	• No evidence of CNV	• Type 1 CNV	• Type 1 CNV with aneurysm or polypoidal lesions	• No evidence of CNV/early type 1 CNV • Inner choroidal flow signal attenuation zones, mostly related with pachyvessels
AF	N/A	• Hyperautofluorescence and/or hypoautofluorescence related to RPE changes	• Hyperautofluorescence and/or hypoautofluorescence related to RPE changes • Vertical, gravitational tracts of RPE hypopigmentation	• Mottled autofluorescence or granular transmission hyperfluorescence or gravitational tracks	• Absence of zonal areas of hyperautofluorescence or hypoautofluorescence and gravitational descending tracts of fundus AF abnormalities		• Variable hyperautofluorescence and hypoautofluorescence of the pigmentary lesions • Absence of oblong, vertical, gravitational tracts of RPE hypopigmentation or geographic areas of speckled hyperautofluorescence indicative of previous SRF
FA	N/A	• Varying degrees of hyperfluorescence and hypofluorescence related to the RPE alterations • Hyperfluorescence corresponding to transmission defects associated with RPE attenuation without leakage in the mid- or late-phase FA • Absence of leakage unless complicated by CNV or CSC	• Leaks at the level of RPE • Ink-blot or smokestack leakage pattern • Multiple indistinct leaks	• Hyperfluorescent staining in a ring-like configuration immediately surrounding the optic disc with mild diffuse leakage	• Hyperfluorescent leakage from CNV	• Usually appear as occult CNV. Classic CNV pattern can be seen in a small proportion of cases	
ICGA	• Choroidal hyperpermeability • Choroidal venous dilation and vascular congestion	• Early hypofluorescence around the FCE or in areas outside the FCE, suggesting filling defects • Prominent dilated choroidal vessels (pachyvessels) coursing around the area of FCE, sometimes on opposite sides of the lesion, seen best in the early to mid phase • Punctate, patchy, or diffuse hyperfluorescence around the FCE in the late phase		• Peripapillary dilated choroidal vessels with multifocal hyperpermeability		• Single or multiple polyps can be seen in the early phase of ICGA • Branching vascular network with terminal aneurysmal dilatations	

*AF* autofluorescence, *CNV* choroidal neovascularization, *CSC* central serous chorioretinopathy, *ELM* external limiting membrane, *EZ* ellipsoid zone, *FA* fluorescein angiography, *FCE* focal choroidal excavation, *ICGA* indocyanine green angiography, *N/A* not applicable, *OCT* optical coherence tomography, *OCT-A* optical coherence tomography angiography, *ONL* outer nuclear layer, *PCV* polypoidal choroidal vasculopathy, *PED* pigment epithelial detachment, *PNV* pachychoroid neovasculopathy, *PPE* pachychoroid pigment epitheliopathy, *PPS* peripapillary pachychoroid syndrome, *RPE* retinal pigment epithelium, *SRF* subretinal fluid

### Quantitative features

#### Congestion as manifested by thickening on OCT

Initially, the term “pachychoroid” was used to describe eyes with increased choroidal thickness compared with normal eyes (Fig. 1f). However, the normative value of choroidal thickness is influenced by multiple factors (e.g., age, axial length, blood pressure), making it difficult to define what is normal. Some investigators have therefore tried to establish a quantitative threshold for defining an eye as having a thick choroid. Previously considered cut-off values for a thick choroid include > 300-μm subfoveal thickness, exceeding subfoveal choroidal thickness by 50 μm, > 347-μm subfoveal thickness, and over 390-μm subfoveal thickness; however, there is no universally accepted threshold value to date [20–22]. Indeed, the presence of retinal disease increases the variability of choroidal thickness measurements between OCT devices [23, 24]. Although choroidal thickness measurements in eyes with pachychoroid disease are comparable between swept-source OCT and spectral domain OCT with enhanced-depth imaging (EDI) devices, in some cases, such as when drusen or subretinal fluid are present, the choroid-scleral border may be unclear with spectral domain OCT. Evidence suggests that swept-source OCT may be more suitable for observation and follow-up of choroidal structures in eyes with choroidal thickness > 400 μm and subfoveal active lesions [23]. The presence of a larger stroma area in the inner choroid has been found via EDI-OCT and is thought to be related to inflammation and edema [25]. Nevertheless, this feature still needs to be verified to discover whether it is applicable to the whole pachychoroid spectrum.

#### Choroidal hyperpermeability

The relative opacity of the retinal pigment epithelium (RPE) to visible light prevents visualization of the choroid by FA (Fig. 1a–d). However, the greater transparency of the RPE to longer wavelengths and the capacity of indocyanine green to bind to the albumin enable choroidal vessels to be delineated better by ICGA. Using ICGA to image disorders included within the pachychoroid spectrum can reveal delayed choroidal filling, choroidal venous dilatation, and hyperfluorescence [26–28]. While the exact mechanism of ICGA hyperfluorescence remains uncertain, one hypothesis is that it is a functional consequence of choroidal ischemia, which in turn results from congestion and stasis within a choroidal lobule [26–28].

Ersoz et al. [10] considered choroidal hyperpermeability to be hyperfluorescent areas with an indistinct margin observed from 7 to 15 min of ICGA. After comparison of eyes uncomplicated by pathological findings such as subretinal fluid, CNV, and RPE atrophy (“uncomplicated pachychoroid”) to eyes with CSC or PPE, Ersoz et al. concluded that choroidal hyperpermeability is the main difference between complicated and uncomplicated pachychoroid. Choroidal hyperpermeability was present in 50% of the patients with uncomplicated pachychoroid, compared with 92% and 93% of patients with PPE and CSC, respectively. No differences were found in subfoveal choroidal thickness and choroidal vessel density [10].

#### Blood-flow signal attenuation within the choriocapillaris and inner choroid with OCT angiography

In pachychoroid disease, the spatial distribution of RPE changes, neurosensory detachment, and neovascularization seem to correlate with the localized choroidal thickening attributable to dilatation of Haller’s layer vessels (Fig. 1e) and thinning of the choriocapillaris (Fig. 1g ) and Sattler’s layers. However, no significant differences in choroidal thickness or vessel density with traditional FA or ICGA have been found between eyes with uncomplicated pachychoroid and eyes on the pachychoroid pathological spectrum [10].

Quantification of choriocapillaris flow characteristics with OCT angiography (OCT-A) has been addressed by several investigators. Some have searched for functional changes at the level of the innermost part of the choriocapillaris, with a working hypothesis that a reduction in flow signal might be related anatomically to structural sequelae [14, 29–31]. Gal-Or et al. [14] found a higher prevalence of inner choroidal flow signal attenuation zones in pachychoroid eyes than in normal controls, and most (68%) of these zones were related anatomically to pachyvessels. Although the mechanisms by which choriocapillaris flow characteristics can contribute to structural sequelae remain elusive, and the determination of whether they are precursors to choriocapillaris thinning would require longitudinal analysis, it is possible that they represent zones of inner choroidal ischemia [14]. Similarly, other studies have suggested the idea of a primary choroidopathy, where microvascular flow deficits may constitute one of the underlying subclinical changes preceding pachychoroid spectrum disorders [30].

### Qualitative features

#### Pachyvessels or dilated choroidal vessels in Haller’s layer

**Fig. 2:**
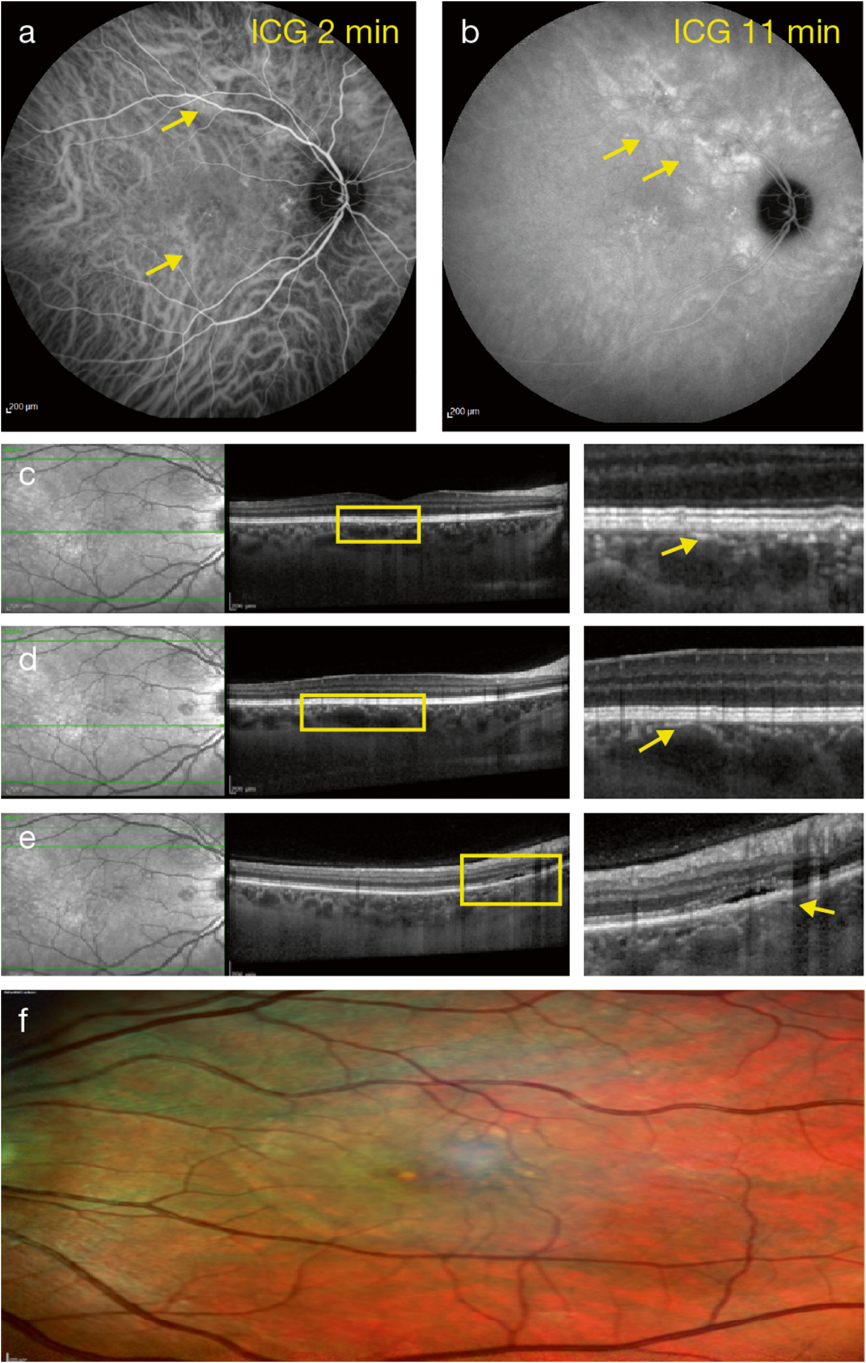
Right eye of a patient with CSC with mid-phase ICGA hyperfluorescence (**a**, **b**), pachyvessels (**c**, **d**), and pigment epithelium detachment (**e**). Multicolor imaging showing pachydrusen (**f**). CSC, central serous chorioretinopathy; ICGA, indocyanine green angiography

This can be the most salient sign within the pachychoroid phenotype. Some investigators have identified these vessels as groups of diagonally orientated hyporeflective Haller’s layer features, with club-shaped origins at the posterior pole and a large and constant caliber for the entire length of the vessel [14]. However, the lack of a cut-off value for defining the large caliber vessels makes their detection subjective. Pachyvessels or hypertrophic vascular entities are thought to result in focal or diffuse increased choroidal thickening and overlying choriocapillaris, with Sattler’s layer thinning and its asymmetry, as revealed by en face OCT projection, being a common finding within the spectrum (Fig. 2c–e) [32, 33].

#### RPE layer alterations and thinning of outer nuclear layer

RPE alterations in eyes with pachychoroid disease are associated with ICGA hyperfluorescence (Fig. 2a–b) and could be colocalized and attributed putatively to foci of choroidal thickening over pachyvessels directly external to the RPE/Bruch’s membrane complex [1, 18]. Focal attenuation of choriocapillaris and Sattler’s layers overlying pachyvessels brings them closer to the Bruch–RPE complex. The inward displacement of these congestive pachyvessels and atrophy of the choriocapillaris may lead to disruption of the RPE and Bruch’s membrane due to ischemia (seen as delayed arterial filling of the choroid on FA and ICGA or as blood-flow signal attenuation within the choriocapillaris and inner choroid with OCT-A) [14, 30] and/or chronic trauma caused by increased choroidal pulsation [34]. A thinner outer nuclear layer has also been described in eyes with pigment epitheliopathy, possibly related to focal ischemia of the choriocapillaris, leading to RPE disruption and photoreceptor apoptosis (Fig. 2c–e) [18].

Differences between eyes in terms of resistance of the RPE and Bruch’s membrane to choroidal pressure may lead to the development of different forms of pachychoroid disease [18]. On fundus autofluorescence, RPE alterations may vary depending on the absence of history of subretinal fluid. In PPE, hyperautofluorescence and hypoautofluorescence of the pigmentary lesions, with or without adjacent areas of granular hypoautofluorescence, have been noted. In patients with previous subretinal fluid, zonal areas of diffuse hyperautofluorescence, hypoautofluorescence, or descending tracts of fundus autofluorescence abnormalities are a common finding [1].

#### Choroidal neovascularization

CNV is a proliferative vascular complication encountered during the natural course of degenerative, dystrophic, traumatic, inflammatory, and malignant chorioretinal diseases [35]. CNV is commonly classified based on the anatomical position of the neovascular complex relative to the RPE, as determined by multimodal in vivo imaging. The mechanism by which pachychoroid might predispose to type 1 neovascularization (characterized by the growth of vessels in the sub-RPE compartment above Bruch’s membrane) or type 2 neovascularization (characterized by the growth of vessels between the RPE and the retina) [36] is as yet undetermined, but relative ischemia due to choriocapillaris attenuation and/or chronic mechanical disruption of Bruch’s membrane has been proposed [35, 37].

#### Pachydrusen

Pachydrusen is a subtype of drusen that is usually associated with increased choroidal thickness, choroidal hyperpermeability, and underlying pachyvessels (Fig. 2f) [38–41]. Drusen are found in the context of surrounding pigmentary changes and are distributed throughout the posterior pole. Pachydrusen are typically > 125 μm in diameter, present either in isolation or in groups of only a few yellow-white deposits, and have well-defined outer borders where the outer border is not necessarily a smooth convex curve but can demonstrate a jagged margin around an elevated subretinal pigment epithelial accumulation [41].

## Clinical spectrum of disorders

Pachychoroid constitutes a common pathogenic process, and within the clinical spectrum, overlapping features [19] and progression from one disorder to another are frequently observed. Several disorders are currently included within the spectrum and, according to their main form of presentation, can be subdivided into three subtypes: (a) those with exudative changes, (b) those that develop neovascularization, and (c) those with atrophic changes.

### Disorders with exudative changes

#### Focal choroidal excavation (FCE)

**Fig. 3:**
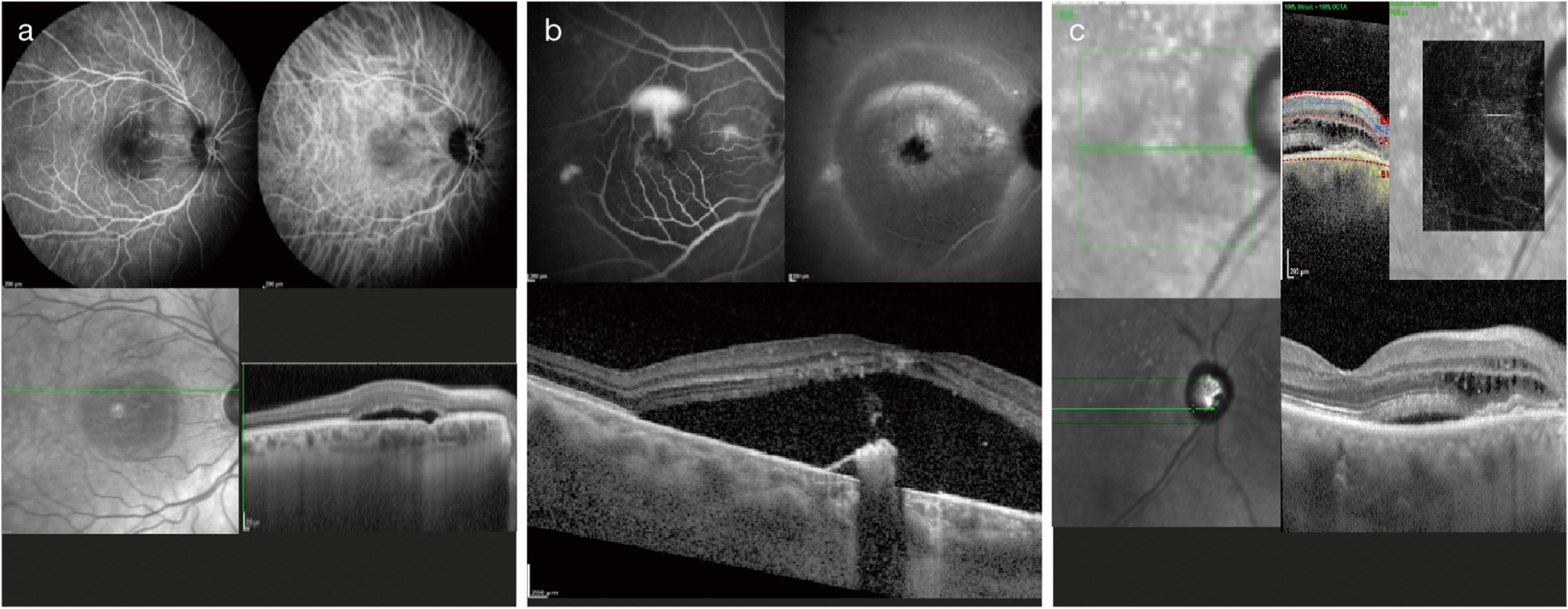
Clinical spectrum of the pachychoroid phenotype. Focal choroidal excavation (**a**), central serous chorioretinopathy (**b**), and peripapillary pachychoroid syndrome (**c**)

FCE is a structural abnormality with an unknown etiology, consistent with a localized concavity in the choroid occurring without posterior staphyloma, trauma, or scleral ectasia (Fig. 3a). Pachychoroid features in eyes with FCE include hyperfluorescence with localized choriocapillaris thinning on ICGA [12]. Fundus examination may appear normal or show nonspecific pigmentary changes or indistinct yellow-whitish spots in an area of reduced fundus tessellation [42]. FCE has been divided into two patterns according to OCT findings: nonconforming FCE, where the photoreceptor tips are separated from the RPE, and conforming FCE, where there is no separation between the photoreceptors and RPE and it is detectable on spectral domain OCT [42].

#### Central serous chorioretinopathy

CSC is a retinal disorder occurring in otherwise healthy young or middle-aged patients who develop serous detachment of the neurosensory retina, often associated with underlying serous elevation of the RPE (Fig. 3b) [1]. It is believed that choroidal congestion and choroidal hyperpermeability, which manifest as increased choroidal thickness and dilated choroidal vessels on EDI-OCT, play a contributing role in serous pigment epithelial detachment and lead to focal defects of the RPE and subretinal fluid most commonly located in the macular region [1]. The chronic presence of subretinal fluid can ultimately damage the RPE, although in some cases, the underlying multifocal choroidal vascular dysfunction can directly affect the RPE without the presence of subretinal fluid [43]. To date, no consensus has been reached on the classification of CSC, and with respect to the time course, acute, subacute, and chronic forms have been described, as well as focal and multifocal forms with respect to their special distribution [44].

#### Peripapillary pachychoroid syndrome

Peripapillary pachychoroid syndrome is a newly described entity in which qualitative and quantitative features of pachychoroid are located predominantly in the peripapillary area (Fig. 3c). This disorder presents with peripapillary choroidal thickening associated with intraretinal and/or subretinal fluid extending from the temporal disc margin into the macula or in the nasal side of the disc, in the absence of neovascularization. It shares common features with chronic CSC, such as RPE alterations, similar ICGA findings, and, in some cases, the presence of serous pigment epithelial detachment, gravitational tracks, evidence of serous retinal detachment outside the peripapillary region with fundus autofluorescence, and outer retinal atrophy. However, the choroidal thickness profile is significantly different from typical CSC in that the nasal macular choroid can be thicker and the thickness can sharply decrease towards the temporal side. Additionally, in peripapillary pachychoroid syndrome, the presence of choroidal folds, older age, and a small cup-to-disc ratio, with mild disc leakage on late FA, are common [11].

### Disorders with neovascularization

#### Pachychoroid neovasculopathy

**Fig. 4:**
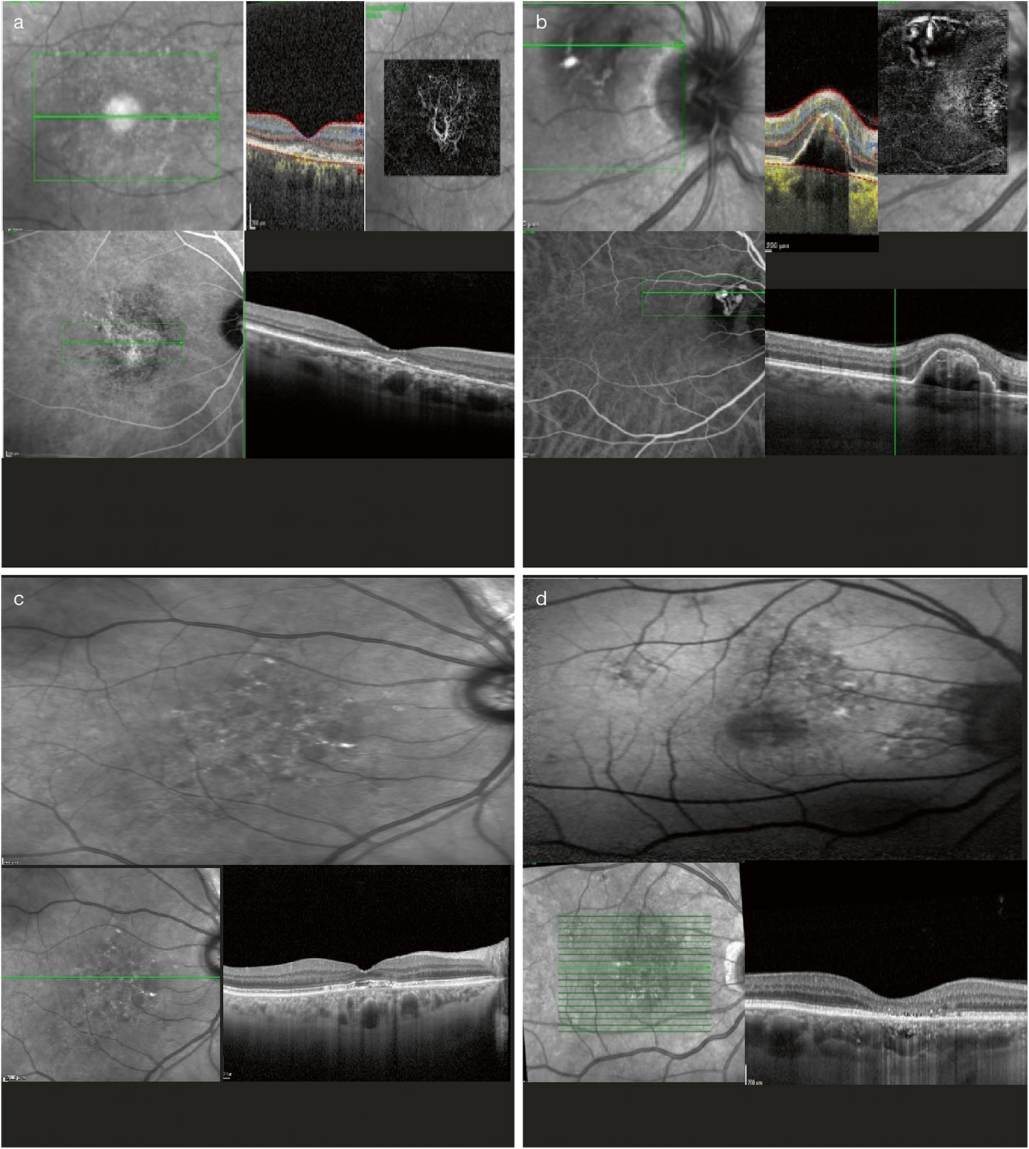
Clinical spectrum of the pachychoroid phenotype. Those with neovascularization: PNV (**a**) and PCV (**b**); and those with atrophy: PPE (**c**) and pachychoroid atrophy (**d**). PCV, polypoidal choroidal vasculopathy; PNV, pachychoroid neovasculopathy; PPE, pachychoroid pigment epitheliopathy

In PNV (Fig. 4a), patients with no evidence of AMD (i.e., with no drusen or only hard drusen), myopic degeneration, or other causes of degeneration develop type 1 neovascular tissue overlying focal areas of choroidal thickening and ICGA hyperfluorescence [45].

#### Polypoidal choroidal vasculopathy

PCV is also known by some investigators as aneurysmal type 1 neovascularization [46] and was first described by Yannuzzi et al. [47] in 1990. It is a disorder that often presents clinically as subretinal pigment RPE and/or subretinal hemorrhage and fluid accumulation, similar to exudative AMD (Fig. 4b). However, recent studies using EDI-OCT and swept-source OCT have demonstrated that patients with PCV/aneurysmal type 1 neovascularization frequently have thick choroids; this is in contrast to the choroidal thinning often observed in eyes with typical nAMD [48]. In 2012, the EVEREST study [49] proposed several diagnostic criteria based on ICGA imaging. According to the study, PCV was diagnosed based on early subretinal ICGA hyperfluorescence (appearing within the first 5 min of ICG dye injection) and at least one of the following diagnostic criteria: (a) nodular appearance of the polypoidal lesion on stereoscopic viewing, (b) hypofluorescent halo around the nodule, c) abnormal vascular channel(s) supplying the polypoidal lesion, (d) pulsatile filling of polypoidal lesion, (e) orange subretinal nodules corresponding to the hyperfluorescent area on ICGA, or (f) massive submacular hemorrhage. Adding multimodal imaging increases the accuracy of the diagnosis and it is possible to see PCV as a variant of type 1 neovascularization, with vascular dilations and a feeding vascular network underneath the RPE [46].

### Disorders with atrophic changes

#### Pachychoroid pigment epitheliopathy

PPE was described by Warrow et al. [1] in 2013 as involving the presence of choroidal congestion and choroidal hyperpermeability. It manifests as increased choroidal thickness and pachyvessels and leads to drusenoid RPE changes and/or small pigment epithelial detachments in patients without the serous macular detachment typical of classic CSC and who also had no history of subretinal fluid (Fig. 4c). Those pigment epithelial detachments can be found directly overlying localized areas of thickened choroid and/or dilated choroidal vessels, and the typical funduscopic appearance of reduced fundus tessellation and FA abnormalities corresponding to RPE disturbances can also be found. As part of the pachychoroid spectrum, subretinal fluid can develop with PPE, similar to CSC (as a *forme fruste* of it), as well as sub-RPE neovascularization with or without polypoidal structures [1] or retinal or RPE choroidal atrophy (Fig. 4d).

## Differential diagnosis

Other diseases involving uveitis (e.g., Vogt–Koyanagi–Harada disease, posterior scleritis, and choroidal granulomas secondary to infectious or inflammatory diseases such as tuberculosis and sarcoidosis) and infiltrative diseases including intraocular lymphomas and intraocular tumors (choroidal hemangioma, choroidal metastases) that also manifest as thickening of the choroid need to be included in the differential diagnosis. However, the presence of other pachychoroid features, in the absence of systemic conditions and signs of intraocular or scleral inflammation, would support the diagnosis of pachychoroid disease.

## Management of neovascular complications of pachychoroid disorders

The EVEREST study was the first to include a set of well-defined ICGA criteria for PCV diagnosis and to assess the effects of verteporfin photodynamic therapy (vPDT) combined with intravitreal ranibizumab (IVR) or vPDT alone versus IVR monotherapy in patients with symptomatic macular PCV. This multicenter study’s primary endpoint was the proportion of patients with ICGA-assessed complete regression of polyps at month 6. After evaluating 61 patients, the study concluded that vPDT combined with IVR or vPDT alone was superior to IVR monotherapy in achieving complete regression of polyps [49]. The EVEREST II study [50], which was conducted a few years later, compared IVR monotherapy with IVR plus adjunctive vPDT administered at baseline. It reported rates of complete polypoidal lesion regression of 33.8% versus 69.7%, respectively, and gains in best-corrected visual acuity (BCVA) from baseline to week 52 of 5.1 letters versus 8.3 letters (*p* = 0.01), respectively. This demonstrates that combining ranibizumab with PDT achieved, on average, significantly greater BCVA gains than ranibizumab monotherapy. The Fujisan study [51] tried to elucidate whether vPDT should be administered at the beginning of treatment or during follow-up of IVR therapy. It found that while both initial and deferred vPDT combined with IVR resulted in similar visual and anatomical improvements at 12 months, the initial vPDT combination led to significantly fewer additional treatments. The LAPTOP study [52] considered as the primary outcome the proportion of patients gaining or losing more than 0.2 logarithm of the minimal angle of resolution (logMAR) units from baseline. In the vPDT arm (*n* = 47), 17.0% achieved visual acuity (VA) gains, 55.3% had no change, and 27.7% experienced VA losses, compared with 30.4%, 60.9%, and 8.7%, respectively, in the ranibizumab arm (*n* = 46). The better VA in the initial IVR group compared with the vPDT group at 2 years was retained at the 5-year follow-up [53]. Regarding aflibercept, the PLANET study [39] aimed to evaluate the efficacy, safety, and tolerability of intravitreal aflibercept injection (IAI) monotherapy versus IAI plus rescue vPDT in the treatment of PCV. Compared with EVEREST II, this study had methodological differences, such as in the role of vPDT (adjunct vs. rescue in EVEREST II vs. PLANET), timing of vPDT administration (from baseline vs. deferred after 3 months), and anti-vascular endothelial growth factor (VEGF) dosing (*pro re nata* vs. fixed). Nevertheless, an improvement in visual and/or functional outcomes was achieved in > 85% of participants treated with IAI monotherapy, with no signs of leakage from polypoidal lesions in > 80% of participants. After week 52, treatment intervals could be extended beyond 8 weeks at investigators’ discretion, and results at week 96 showed that IAI monotherapy was noninferior to IAI plus rescue vPDT in terms of ETDRS letters gained (+ 10.7 vs. + 9.1; *p* = 0.48) and the proportions of patients with complete polyp regression (33.1% vs. 29.1%) or without active polyps (82.1% vs. 85.6%) [54]. These results may suggest differences in the treatment effects of IAI versus ranibizumab in individuals with PCV.

There is no large randomized controlled study for the treatment of PNV. Some studies suggest anti-VEGF monotherapy is generally effective. For example, Matsumoto et al. [55] have shown that a treat-and-extend regimen of intravitreal anti-VEGF injection was equally effective in terms of improvement in BCVA and exudative changes, both in eyes with PNV and type 1 nAMD. However, further studies are needed to investigate whether PDT monotherapy or combination therapy (anti-VEGF plus PDT) is more effective than anti-VEGF monotherapy.

## Management of pachychoroid disorders with exudative changes

Several treatment strategies have been described for the management of exudative forms of pachychoroid disease in the absence of neovascularization.

Traditionally, the treatment of choice for CSC has been focal continuous-wave thermal laser treatment, typically with an argon or diode laser. Although thermal laser treatment can reduce the duration of subretinal fluid, the final BCVA does not differ significantly compared with no treatment, and its use should be limited to extrafoveal leakage sites. Other therapies include transpupillary thermotherapy using an 810-nm pulse diode laser with a treatment duration of 30–45 s, a subthreshold micropulse laser, and the use of antagonists of mineralocorticoid receptors and glucocorticoid receptors, among other systemic treatments. However, in recent years, vPDT has emerged to become the treatment of choice for many retina specialists. vPDT may be performed using either full- or half-dose verteporfin, and the light irradiance may be applied using either half-fluence or one-half the standard irradiation time. The PLACE (half-dose photodynamic therapy versus high-density subthreshold micropulse laser treatment in patients with chronic central serous chorioretinopathy) trial, a large, prospective, multicenter, randomized-controlled treatment trial for the treatment of chronic CSC, showed superiority in terms of complete resolution of subretinal fluid and functional improvement of half-dose vPDT versus high-density subthreshold micropulse laser [56]. This review summarized important options for the treatment of CSC. However, treatment for CSC is still controversial. For interested readers, a recent extensive review is recommended [43].

## Discussion

Initially, pachychoroid was considered only as increased choroidal thickness compared with normal eyes. The wide range of choroidal thicknesses considered normal is influenced by multiple factors (age, axial length, blood pressure), and there is no definitive cut-off value to date. Additionally, while a thick choroid is frequently seen, choroidal thickness per se is not the most important criterion for defining the pachychoroid disease phenotype. Other diseases involving uveitis, such as Vogt–Koyanagi–Harada disease, and intraocular tumors like choroidal hemangioma also result in a thickened choroid and are not considered as being on the pachychoroid spectrum due to having different etiology. This highlights the idea that the identification of other choroidal morphological alterations is critical for the appropriate diagnosis of pachychoroid disease. Moreover, choroidal thickness may be dependent on the duration (chronicity) of the disease process. With longer patient follow-up, we may find that pachychoroid spectrum eyes with initially thick choroids end up with thinner choroids due to atrophy. Longitudinal studies are therefore needed to investigate whether the decrease in choroidal thickness is due to the natural history of pachychoroid or is in response to treatment (intravitreal treatment [57] or vPDT [58]).

Our literature review suggests that the presence of dilated or congested vessels in the choroid, not thickened choroid per se, is the most salient feature of pachychoroid. Eyes with pachychoroid but without exudation, CNV, or RPE atrophy (uncomplicated pachychoroid) have choroidal hyperpermeability, focal or diffuse congestion, or an increase in choroidal thickness and pachyvessels [10]. Pachyvessels are thought to result in focal or diffuse increased choroidal thickening, overlying choriocapillaris, and Sattler’s layer thinning [32]. The inward displacement of the pachyvessels and the reduced thickness and decreased flow of the choriocapillaris may lead to the disruption of the RPE and Bruch’s membrane due to ischemia [14, 30] and/or chronic trauma caused by increased choroidal pulsation [34] or mechanical stress. Variable resistance of the RPE to fluid/hydrostatic pressure imbalance and the different degrees of ischemia and inflammation with the consecutive development of choroidal neovascularization may lead to the distinct disorders of the pachychoroid spectrum.

Studies by Koizumi et al. [59] and Takahashi et al. [60], which compared subfoveal choroidal structures among patients with typical AMD and PCV, have suggested that the intrachoroidal structures of eyes with typical nAMD and PCV differ significantly. In eyes with PCV, there seemed to be greater dilation of the large choroidal vessels compared with eyes with nAMD; this morphologic difference may be a surrogate for differences in the pathophysiology and may explain differences in treatment outcomes observed with PCV compared with exudative AMD [7].

Describing the epidemiological, clinical, and morphological characteristics of each disorder encompassed on the pachychoroid spectrum is essential to disease monitoring, prognosis, and treatment decisions. For example, in PCV, several morphological characteristics may influence the prognosis. Choroidal morphology, including characteristic features of pachychoroid and high vascularity, can serve as predictive factors for outcomes after combination PDT [40] or aflibercept [61–63]. Following IAI, greater improvements in VA at 12 months were significantly associated with greater baseline luminal area and smaller baseline stromal area in PCV [61]. Patients with PCV associated with choroidal hyperpermeability more frequently demonstrated bilateral CNV, a history of CSC, a thickened choroid, and poor responses to IVR than those without choroidal hyperpermeability [64]. Regarding PNV, distinct aqueous humor cytokine profiles of patients with PNV and nAMD have been described, suggesting that angiogenic factors and proinflammatory cytokines may play distinct roles in the pathogenesis of PNV and nAMD [65].

## Conclusion

A consensus on pachychoroid terms is needed. The presence of focally dilated or congested vessels in the choroid, not thickened choroid per se, with overlying choriocapillaris thinning in the posterior pole is the most salient feature of pachychoroid. However, other qualitative and quantitative features should also be taken into account in order to differentiate the pathological pachychoroid clinical spectrum. Clinicians should be aware of the different disorders encompassed on the spectrum and of their main forms of presentation, which can be subdivided into those with exudation, those with neovascularization, and those with RPE and chorioretinal atrophic changes. Choroidal features would be useful in the management of patients, and in terms of prognosis, characteristic features of pachychoroid may serve as a predictive factor, but these are still to be confirmed in a large study.

## References

[1] Warrow DJ, Hoang QV, Freund KB (2013). Pachychoroid pigment epitheliopathy. Retina.

[2] Mrejen S, Spaide RF (2013). Optical coherence tomography: imaging of the choroid and beyond. Surv Ophthalmol.

[3] Miyake M, Ooto S, Yamashiro K, Takahashi A, Yoshikawa M, Akagi-Kurashige Y, Ueda-Arakawa N, Oishi A, Nakanishi H, Tamura H, Tsujikawa A, Yoshimura N (2015). Pachychoroid neovasculopathy and age-related macular degeneration. Sci Rep.

[4] Wong WL, Su X, Li X, Cheung CMG, Klein R, Cheng C-Y, Wong TY (2014). Global prevalence of age-related macular degeneration and disease burden projection for 2020 and 2040: a systematic review and meta-analysis. Lancet Glob Health.

[5] Wong CW, Yanagi Y, Lee W-K, Ogura Y, Yeo I, Wong TY, Cheung CMG (2016). Age-related macular degeneration and polypoidal choroidal vasculopathy in Asians. Prog Retin Eye Res.

[6] Fujiwara K, Yasuda M, Hata J, Oshima Y, Hashimoto S, Yoshitomi T, Kiyohara Y, Ishibashi T, Ninomiya T, Sonoda K-H (2018). Prevalence and risk factors for polypoidal choroidal vasculopathy in a general Japanese population: the Hisayama study. Semin Ophthalmol.

[7] Li Y, You QS, Wei WB, Xu J, Chen CX, Wang YX, Xu L, Jonas JB (2014). Polypoidal choroidal vasculopathy in adult Chinese: the Beijing Eye Study. Ophthalmology.

[8] Li Y, You QS, Wei WB, Xu J, Chen CX, Wang YX, Xu L, Jonas JB (2016). Prevalence and associations of central serous chorioretinopathy in elderly Chinese. The Beijing Eye Study 2011. Acta Ophthalmol.

[9] Kitzmann AS, Pulido JS, Diehl NN, Hodge DO, Burke JP (2008). The incidence of central serous chorioretinopathy in Olmsted County, Minnesota, 1980-2002. Ophthalmology.

[10] Ersoz MG, Arf S, Hocaoglu M, Sayman Muslubas I, Karacorlu M (2018). Indocyanine green angiography of pachychoroid pigment epitheliopathy. Retina.

[11] Phasukkijwatana N, Freund KB, Dolz-Marco R, Al-Sheikh M, Keane PA, Egan CA, Randhawa S, Stewart JM, Liu Q, Hunyor AP, Kreiger A, Nagiel A, Lalane R, Rahimi M, Lee WK, Jampol LM, Sarraf D (2018). Peripapillary pachychoroid syndrome. Retina.

[12] Luk FOJ, Fok ACT, Lee A, Liu ATW, Lai TYY (2015). Focal choroidal excavation in patients with central serous chorioretinopathy. Eye (Lond).

[13] Chung H, Byeon SH, Freund KB (2017). Focal choroidal excavation and its association with pachychoroid spectrum disorders: a review of the literature and multimodal imaging findings. Retina.

[14] Gal-Or O, Dansingani KK, Sebrow D, Dolz-Marco R, Freund KB (2018). Inner choroidal flow signal attenuation in pachychoroid disease: optical coherence tomography angiography. Retina.

[15] Iida T, Yannuzzi LA, Spaide RF, Borodoker N, Carvalho CA, Negrao S (2003). Cystoid macular degeneration in chronic central serous chorioretinopathy. Retina.

[16] Matsumoto H, Sato T, Kishi S (2009). Outer nuclear layer thickness at the fovea determines visual outcomes in resolved central serous chorioretinopathy. Am J Ophthalmol.

[17] Cheung CMG, Lee WK, Koizumi H, Dansingani K, Lai TYY, Freund KB (2019). Pachychoroid disease. Eye (Lond).

[18] Ersoz MG, Karacorlu M, Arf S, Hocaoglu M, Sayman Muslubas I (2018). Outer nuclear layer thinning in pachychoroid pigment epitheliopathy. Retina.

[19] Manayath GJ, Shah VS, Saravanan VR, Narendran V (2018). Polypoidal choroidal vasculopathy associated with central serous chorioretinopathy: pachychoroid spectrum of diseases. Retina.

[20] Dansingani KK, Balaratnasingam C, Naysan J, Freund KB (2016). En face imaging of pachychoroid spectrum disorders with swept-source optical coherence tomography. Retina.

[21] Chen G, Tzekov R, Li W, Jiang F, Mao S, Tong Y (2017). Subfoveal choroidal thickness in central serous chorioretinopathy: a meta-analysis. PLoS One.

[22] Lehmann M, Bousquet E, Beydoun T, Behar-Cohen F (2015). Pachychoroid: an inherited condition?. Retina.

[23] Lee M-W, Park H-J, Shin Y-I, Lee W-H, Lim H-B, Kim J-Y (2020). Comparison of choroidal thickness measurements using swept source and spectral domain optical coherence tomography in pachychoroid diseases. PLoS One.

[24] Tan CSH, Ngo WK, Cheong KX (2015). Comparison of choroidal thicknesses using swept source and spectral domain optical coherence tomography in diseased and normal eyes. Br J Ophthalmol.

[25] Sonoda S, Sakamoto T, Kuroiwa N, Arimura N, Kawano H, Yoshihara N, Yamashita T, Uchino E, Kinoshita T, Mitamura Y (2016). Structural changes of inner and outer choroid in central serous chorioretinopathy determined by optical coherence tomography. PLoS One.

[26] Prünte C, Flammer J (1996). Choroidal capillary and venous congestion in central serous chorioretinopathy. Am J Ophthalmol.

[27] Iida T, Kishi S, Hagimura N, Shimizu K (1999). Persistent and bilateral choroidal vascular abnormalities in central serous chorioretinopathy. Retina.

[28] Kitaya N, Nagaoka T, Hikichi T, Sugawara R, Fukui K, Ishiko S, Yoshida A (2003). Features of abnormal choroidal circulation in central serous chorioretinopathy. Br J Ophthalmol.

[29] Chan SY, Wang Q, Wei WB, Jonas JB (2016). Optical coherence tomographic angiography in central serous chorioretinopathy. Retina.

[30] Rochepeau C, Kodjikian L, Garcia M-A, Coulon C, Burillon C, Denis P, Delaunay B, Mathis T (2018). Optical coherence tomography angiography quantitative assessment of choriocapillaris blood flow in central serous chorioretinopathy. Am J Ophthalmol.

[31] Yun C, Huh J, Ahn SM, Lee B, Kim JT, Hwang S-Y, Kim S-W, Oh J (2019). Choriocapillaris flow features and choroidal vasculature in the fellow eyes of patients with acute central serous chorioretinopathy. Graefes Arch Clin Exp Ophthalmol.

[32] Lee WK, Baek J, Dansingani KK, Lee JH, Freund KB (2016). Choroidal morphology in eyes with polypoidal choroidal vasculopathy and normal or subnormal subfoveal choroidal thickness. Retina.

[33] Hiroe T, Kishi S (2018). Dilatation of asymmetric vortex vein in central serous chorioretinopathy. Ophthalmol Retina.

[34] Tittl M, Polska E, Kircher K, Kruger A, Maar N, Stur M, Schmetterer L (2003). Topical fundus pulsation measurement in patients with active central serous chorioretinopathy. Arch Ophthalmol.

[35] Balaratnasingam C, Lee WK, Koizumi H, Dansingani K, Inoue M, Freund KB (2016). Polypoidal choroidal vasculopathy: a distinct disease or manifestation of many?. Retina.

[36] Tamura H, Tsujikawa A, Otani A, Gotoh N, Sasahara M, Kameda T, Iwama D, Yodoi Y, Mandai M, Yoshimura N (2007). Polypoidal choroidal vasculopathy appearing as classic choroidal neovascularisation on fluorescein angiography. Br J Ophthalmol.

[37] Lee J, Kim M, Lee CS, Kim SS, Koh HJ, Lee SC, Byeon SH (2020). Drusen subtypes and choroidal characteristics in Asian eyes with typical neovascular age-related macular degeneration. Retina.

[38] Lee J, Byeon SH (2019). Prevalence and clinical characteristics of pachydrusen in polypoidal choroidal vasculopathy: multimodal image study. Retina.

[39] Lee WK, Iida T, Ogura Y, Chen SJ, Wong TY, Mitchell P, Cheung GCM, Zhang Z, Leal S, Ishibashi T, PLANET Investigators (2018) Efficacy and safety of intravitreal aflibercept for polypoidal choroidal vasculopathy in the PLANET study: a randomized clinical trial. JAMA Ophthalmol 136:786–793. 10.1001/jamaophthalmol.2018.1804 10.1001/jamaophthalmol.2018.1804 PMC6136040 29801063

[40] Baek J, Lee JH, Jeon S, Lee WK (2019). Choroidal morphology and short-term outcomes of combination photodynamic therapy in polypoidal choroidal vasculopathy. Eye (Lond).

[41] Spaide RF, Fujimoto JG, Waheed NK (2015). Image artifacts in optical coherence tomography angiography. Retina.

[42] Margolis R, Mukkamala SK, Jampol LM, Spaide RF, Ober MD, Sorenson JA, Gentile RC, Miller JA, Sherman J, Freund KB (2011). The expanded spectrum of focal choroidal excavation. Arch Ophthalmol.

[43] van Rijssen TJ, van Dijk EHC, Yzer S, Ohno-Matsui K, Keunen JEE, Schlingemann RO, Sivaprasad S, Querques G, Downes SM, Fauser S, Hoyng CB, Piccolino FC, Chhablani JK, Lai TYY, Lotery AJ, Larsen M, Holz FG, Freund KB, Yannuzzi LA, Boon CJF (2019). Central serous chorioretinopathy: towards an evidence-based treatment guideline. Prog Retin Eye Res.

[44] Singh SR, Matet A, van Dijk EHC, Daruich A, Fauser S, Yzer S, Peiretti E, Sivaprasad S, Lotery AJ, Boon CJF, Behar-Cohen F, Freund KB, Chhablani J (2019). Discrepancy in current central serous chorioretinopathy classification. Br J Ophthalmol.

[45] Pang CE, Freund KB (2015). Pachychoroid neovasculopathy. Retina.

[46] Dansingani KK, Gal-Or O, Sadda SR, Yannuzzi LA, Freund KB (2018). Understanding aneurysmal type 1 neovascularization (polypoidal choroidal vasculopathy): a lesson in the taxonomy of 'expanded spectra' - a review. Clin Exp Ophthalmol.

[47] Yannuzzi LA, Sorenson J, Spaide RF, Lipson B (1990). Idiopathic polypoidal choroidal vasculopathy (IPCV). Retina.

[48] Chung SE, Kang SW, Lee JH, Kim YT (2011). Choroidal thickness in polypoidal choroidal vasculopathy and exudative age-related macular degeneration. Ophthalmology.

[49] Koh A, Lee WK, Chen L-J, Chen S-J, Hashad Y, Kim H, Lai TY, Pilz S, Ruamviboonsuk P, Tokaji E, Weisberger A, Lim TH (2012). EVEREST study: Efficacy and safety of verteporfin photodynamic therapy in combination with ranibizumab or alone versus ranibizumab monotherapy in patients with symptomatic macular polypoidal choroidal vasculopathy. Retina.

[50] Koh A, Lai TYY, Takahashi K, Wong TY, Chen L-J, Ruamviboonsuk P, Tan CS, Feller C, Margaron P, Lim TH, Lee WK, EVEREST II study group (2017) Efficacy and safety of ranibizumab with or without verteporfin photodynamic therapy for polypoidal choroidal vasculopathy: a randomized clinical trial. JAMA Ophthalmol 135:1206–1213. 10.1001/jamaophthalmol.2017.4030 10.1001/jamaophthalmol.2017.4030 PMC5710379 28983556

[51] Gomi F, Oshima Y, Mori R, Kano M, Saito M, Yamashita A, Iwata E, Maruko R, Fujisan Study Group (2015). Initial versus delayed photodynamic therapy in combination with ranibizumab for treatment of polypoidal choroidal vasculopathy: the Fujisan study. Retina.

[52] Oishi A, Kojima H, Mandai M, Honda S, Matsuoka T, Oh H, Kita M, Nagai T, Fujihara M, Bessho N, Uenishi M, Kurimoto Y, Negi A (2013). Comparison of the effect of ranibizumab and verteporfin for polypoidal choroidal vasculopathy: 12-month LAPTOP study results. Am J Ophthalmol.

[53] Miyamoto N, Mandai M, Oishi A, Nakai S, Honda S, Hirashima T, Oh H, Matsumoto Y, Uenishi M, Kurimoto Y (2019). Long-term results of photodynamic therapy or ranibizumab for polypoidal choroidal vasculopathy in LAPTOP study. Br J Ophthalmol.

[54] Wong TY, Ogura Y, Lee WK, Iida T, Chen SJ, Mitchell P, Gemmy Cheung CM, Zhang Z, Leal S, Ishibashi T, PLANET Investigators (2019) Efficacy and safety of intravitreal aflibercept for polypoidal choroidal vasculopathy: two-year results of the aflibercept in polypoidal choroidal vasculopathy study. Am J Ophthalmol 204:80–89. 10.1016/j.ajo.2019.02.027 10.1016/j.ajo.2019.02.027 30849345

[55] Matsumoto H, Hiroe T, Morimoto M, Mimura K, Ito A, Akiyama H (2018). Efficacy of treat-and-extend regimen with aflibercept for pachychoroid neovasculopathy and Type 1 neovascular age-related macular degeneration. Jpn J Ophthalmol.

[56] van Dijk EHC, Fauser S, Breukink MB, Blanco-Garavito R, Groenewoud JMM, Keunen JEE, Peters PJH, Dijkman G, Souied EH, MacLaren RE, Querques G, Downes SM, Hoyng CB, Boon CJF (2018). Half-dose photodynamic therapy versus high-density subthreshold micropulse laser treatment in patients with chronic central serous chorioretinopathy: the PLACE trial. Ophthalmology.

[57] Koizumi H, Kano M, Yamamoto A, Saito M, Maruko I, Sekiryu T, Okada AA, Iida T (2016). Subfoveal choroidal thickness during aflibercept therapy for neovascular age-related macular degeneration: twelve-month results. Ophthalmology.

[58] Izumi T, Koizumi H, Maruko I, Takahashi Y, Sonoda S, Sakamoto T, Iida T (2017). Structural analyses of choroid after half-dose verteporfin photodynamic therapy for central serous chorioretinopathy. Br J Ophthalmol.

[59] Koizumi H, Yamagishi T, Yamazaki T, Kawasaki R, Kinoshita S (2011). Subfoveal choroidal thickness in typical age-related macular degeneration and polypoidal choroidal vasculopathy. Graefes Arch Clin Exp Ophthalmol.

[60] Takahashi A, Ooto S, Yamashiro K, Tamura H, Oishi A, Miyata M, Hata M, Yoshikawa M, Yoshimura N, Tsujikawa A (2018). Pachychoroid geographic atrophy: clinical and genetic characteristics. Ophthalmol Retina.

[61] Daizumoto E, Mitamura Y, Sano H, Akaiwa K, Niki M, Yamanaka C, Kinoshita T, Egawa M, Sonoda S, Sakamoto T (2017). Changes of choroidal structure after intravitreal aflibercept therapy for polypoidal choroidal vasculopathy. Br J Ophthalmol.

[62] Yanagi Y, Mohla A, Lee SY, Mathur R, Chan CM, Yeo I, Wong TY, Cheung CMG (2018). Incidence of fellow eye involvement in patients with unilateral exudative age-related macular degeneration. JAMA Ophthalmol.

[63] Ogasawara M, Koizumi H, Yamamoto A, Itagaki K, Saito M, Maruko I, Okada AA, Iida T, Sekiryu T (2018). Prognostic factors after aflibercept therapy for typical age-related macular degeneration and polypoidal choroidal vasculopathy. Jpn J Ophthalmol.

[64] Koizumi H, Yamagishi T, Yamazaki T, Kinoshita S (2013). Relationship between clinical characteristics of polypoidal choroidal vasculopathy and choroidal vascular hyperpermeability. Am J Ophthalmol.

[65] Terao N, Koizumi H, Kojima K, Yamagishi T, Yamamoto Y, Yoshii K, Kitazawa K, Hiraga A, Toda M, Kinoshita S, Sotozono C, Hamuro J (2018). Distinct aqueous humour cytokine profiles of patients with pachychoroid neovasculopathy and neovascular age-related macular degeneration. Sci Rep.

